# Differences in Bacterial Diversity and Communities Between Glacial Snow and Glacial Soil on the Chongce Ice Cap, West Kunlun Mountains

**DOI:** 10.1038/srep36548

**Published:** 2016-11-04

**Authors:** Guang Li Yang, Shu Gui Hou, Ri Le Baoge, Zhi Guo Li, Hao Xu, Ya Ping Liu, Wen Tao Du, Yong Qin Liu

**Affiliations:** 1 Department of Life Science, Shangqiu Normal University, Shangqiu 476000, China; 2 School of Geographic and Oceanographic Sciences, Nanjing University, Nanjing 210023, China; 3 Department of Environment and Planning, Shangqiu Normal University, Shangqiu 476000, China; 4 Cold and Arid Regions Environmental and Engineering Research Institute, Chinese Academy of Sciences, Lanzhou 730000, China; 5 Key Laboratory of Tibetan Environmental Changes and Land Surface Processes, Institute of Tibetan Plateau Research, Chinese Academy of Sciences, Beijing 100101, China

## Abstract

A detailed understanding of microbial ecology in different supraglacial habitats is important due to the unprecedented speed of glacier retreat. Differences in bacterial diversity and community structure between glacial snow and glacial soil on the Chongce Ice Cap were assessed using 454 pyrosequencing. Based on rarefaction curves, Chao1, ACE, and Shannon indices, we found that bacterial diversity in glacial snow was lower than that in glacial soil. Principal coordinate analysis (PCoA) and heatmap analysis indicated that there were major differences in bacterial communities between glacial snow and glacial soil. Most bacteria were different between the two habitats; however, there were some common bacteria shared between glacial snow and glacial soil. Some rare or functional bacterial resources were also present in the Chongce Ice Cap. These findings provide a preliminary understanding of the shifts in bacterial diversity and communities from glacial snow to glacial soil after the melting and inflow of glacial snow into glacial soil.

In recent years, researchers have proposed the concept of glacial ecology^1^. Three key glacial ecosystems have emerged: the supraglacial, subglacial, and englacial ecosystems, and most studies have focused on supraglacial ecosystems, such as cryoconite^2^, snow^3^, soil^4^, and glacier streams^5^. Moreover, most of these studies have been performed in one single habitat. However, a few studies have examined multiple habitats^6^,^7^,^8^, including glacial runoff and aquatic habitats. These studies have shown that different habitats harbour different microbial communities and biodiversity, albeit with some common groups, even within the same glacier.

Notably, these results with multiple habitats have focused on aquatic environments. In glaciers, when temperatures rise above zero, large volumes of melt water are produced; this melt water first enters glacial soil and then runs into moraine lakes or streams. Glacial soil may represent an important microbial source for the entire supraglacial system since it is connected with glacial snow and moraine lakes/streams. With the continuing glacial retreat, the interactions between bacterial communities in glacial snow and in glacial soil are becoming more significant. However, the bacterial diversity and interactions between communities in glacial snow and in glacial soil proximal to glacial snow are still unclear. Moreover, information regarding unclassified sequences and functional genera in Chongce Ice Cap is still needed. This type of research is necessary to explore changes in microbial resources due to accelerated glacier retreat.

Accordingly, in this study, we aimed to compare the community structures of glacial snow and glacial soil using 454 pyrosequencing of the 16S rRNA gene of microbial samples collected from the Chongce Ice Cap. We hypothesised that snow and soil would harbour different bacterial communities owing to their different environmental features. Moreover, we evaluated certain bacteria that, to the best of our knowledge, have not been reported in other glacial environments. Our study provides a basis for the understanding of microbial ecology in different supraglacial habitats, which is particularly important because of the unprecedented speed of glacier retreat.

## Results and Discussion

A total of 126,728 raw reads and 96,012 operational taxonomic units (OTUs, Table 1) were obtained from the nine samples through 454 pyrosequencing analysis. The average length of the sequences was 478 bp. Each of the nine samples contained between 12,047 and 15,798 raw reads, with OTUs ranging from 7,769 to 13,434 (Table 1).

**Figure 1:**
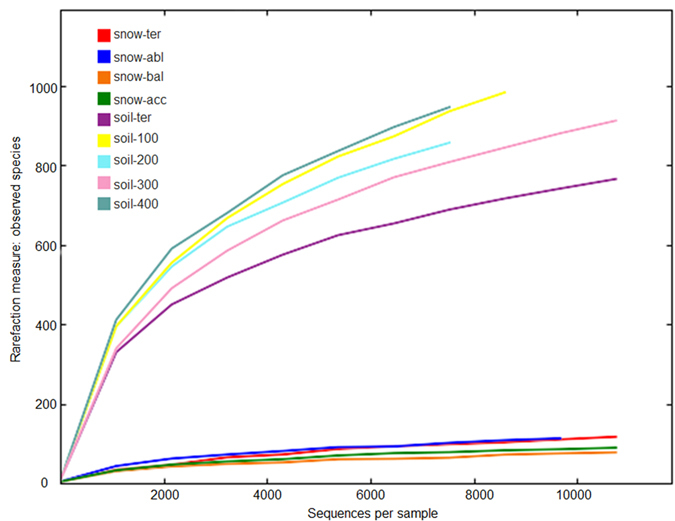
Rarefaction analysis of the nine samples.

The rarefaction curves, based on OTUs at 3% dissimilarity, showed bacterial diversity and richness of the samples (Fig. 1). Rarefaction curves were used to determine whether sufficient sequencing depth was achieved. The glacial snow displayed saturated trends, whereas the glacial soil showed upward trends. In addition, the variations observed for the different curves were supported by the coverage (Table 1) of different samples (95.7–99.7%), indicating that sufficient sequencing depth was obtained in our study.

**Figure 2:**
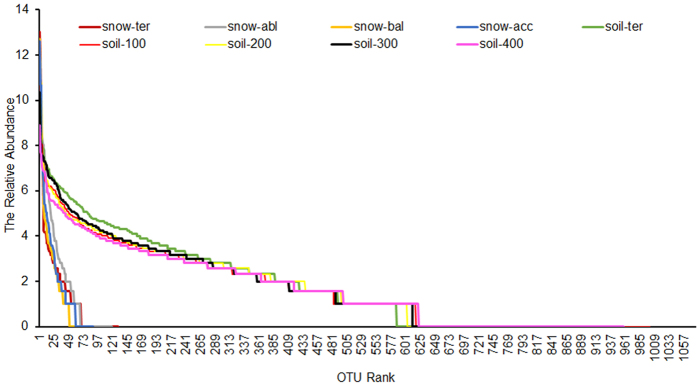
Rank-abundance curve based on bacterial OTUs at a dissimilarity level of 3%.

Rank-abundance curves indicated that the majority of the reads in glacial snow showed more dominance and fewer OTUs, whereas the reads in glacial soil showed the opposite pattern with low dominance and higher richness (Fig. 2).

### Different bacterial diversities between glacial snow and glacial soil

The rarefaction analysis of the OTUs (Fig. 1) indicated that bacterial diversity and richness were higher in glacial soil than in glacial snow. Based on the results of Shannon index analysis, bacterial diversities were 1.175–2.081 in glacial snow (mean 1.568, n = 4) and 5.354–5.892 (mean 5.586, n = 5) in glacial soil (Table 1), consistent with the above rarefaction analysis and the rank-abundance curves. The higher bacterial diversity index in glacial soil than in glacial snow was similar to the study on cryoconite holes and other glacial habitats. Takeuchi *et al*.^9^ showed that cryoconite holes may contain the highest level of biodiversity relative to other glacial habitats. In the Ecology Glacier, Antarctica^10^, functional diversity in Biolog Ecoplates was low in the ice samples, ranging from 1 positive response to 15, and relatively high in cryoconite holes (from 13 to 26). The mean value of Shannon index with DGGE analysis in surface ice was 0.53 (n = 6). A higher bacterial diversity was found in cryoconite holes (mean = 0.68, n = 4). The above results were consistent to the distribution of TOC (3.36 mg/L in ice, 5.55 mg/L in cryoconite), DOC (1.54 mg/L in ice, 2.77 mg/L in cryoconite), POC (1.82 mg/L in ice, 2.79 mg/L in cryoconite), total nitrogen (0.26 mg/L in ice, 1.04 mg/L in cryoconite), total phosphorus (0.05 mg/L in ice, 0.15 mg/L in cryoconite), organic phosphorus (0.02 mg/L in ice, 0.14 mg/L in cryoconite). The results indicated that changing environmental factors influenced the diversity of the microbiocenosis in both habitat types^10^. However, our study is absent of environmental parameters, more studies are still needed to investigate why bacterial diversity in glacial snow is lower than in glacial soil.

The values of the Simpson index were reported as greater than 0 and less than 1. Values near 0 corresponded to highly diverse or heterogeneous ecosystems, and values near 1 corresponded to ecosystems that are more homogeneous. The Simpson index in our study showed a decreasing trend (Table 1) and reflected the increasing heterogeneity of ecosystems from glacial snow to glacial soil.

### Comparison of the bacterial diversity indices with other study sites

The Shannon index in our study was 1.175–2.081 in glacial snow, with an altitude of 5,800–6,100 m. In the Yala glacier, the index was 2.7–3.0^6^, with an altitude of 5,800–6,100 m. In Laohugou No. 12, the Shannon index was 3.83–5.58^11^, with an altitude of 4,271–4,960 m. For glacial soil samples, in our study of the Chongce glacier, the Shannon index was 5.354–5.783, with an altitude of 5,700 m. In Laohugou No. 12, the Shannon index was 6.55, with an altitude of 4,271 m^11^. In Tianshan No. 1, the Shannon index was 6.35–7.55, with an altitude of 3,740–4,480 m^12^. Thus, bacterial diversity index at different study sites was negatively correlated with altitude. Decreased temperature with increasing altitude was the most significant environmental gradient. Effect of temperature on the altitudinal distribution of microbes was also observed by Bahram *et al*.^13^ and Wang *et al*.^14^.

### Different bacterial communities between glacial snow and glacial soil

**Figure 3:**
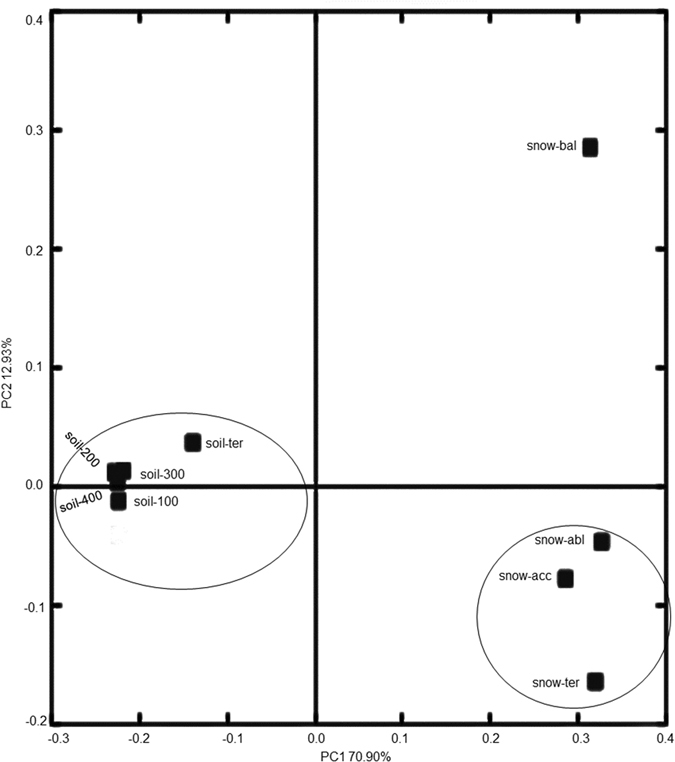
Principal co-ordinate analysis (PCoA) of bacterial communities from the nine samples based on pyrosequencing of the 16S rRNA gene.

To determine the distribution and biogeography of the bacterial community, the 454 pyrosequencing data were analysed in relation to sampling locations using PCoA analysis. As shown in Fig. 3, five soil samples were clustered together and were well separated from the four snow samples. Hierarchy cluster heatmap results (see Supplementary Fig. S1) also showed two clusters: one was from five soil samples, the other was from four snow samples, confirming the above PCoA results. Both PCoA and heatmap analysis highlighted the particular differences in the bacterial communities of glacial snow and glacial soil. The above results were similar to the findings of the Svalbard glacier^8^ and Yala glacier^6^. For the Svalbard glacier, bacterial communities in the snow libraries were significantly different from those of the meltwater libraries. For the Yala glacier, microbial communities in the moraine lakes and glacier streams were completely different from those in snow.

**Figure 4:**
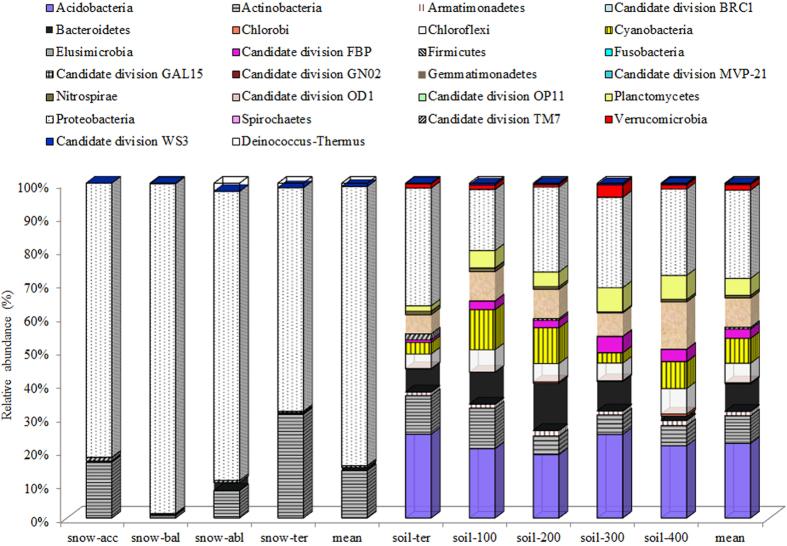
Bacterial community distribution at the phylum level in the nine samples.

Next, 454 pyrosequencing was used to detect 26 phyla from the nine samples (Fig. 4). The proportion of dominant sequence must be above 1% with mean value within 4 snow samples or 5 soil samples. Actinobacteria and Proteobacteria were dominant in both glacial snow and glacial soil, ubiquitous, with extensive metabolic versatility. Additionally, Deinococcus-Thermus were dominant in glacial snow. Whereas, Acidobacteria, Armatimonadetes, Bacteroidetes, Chloroflexi, Cyanobacteria, Candidate division FBP, Gemmatimonadetes, Planctomycetes, Verrucomicrobia were dominant in glacial soil (see Supplementary Table S1).

**Figure 5:**
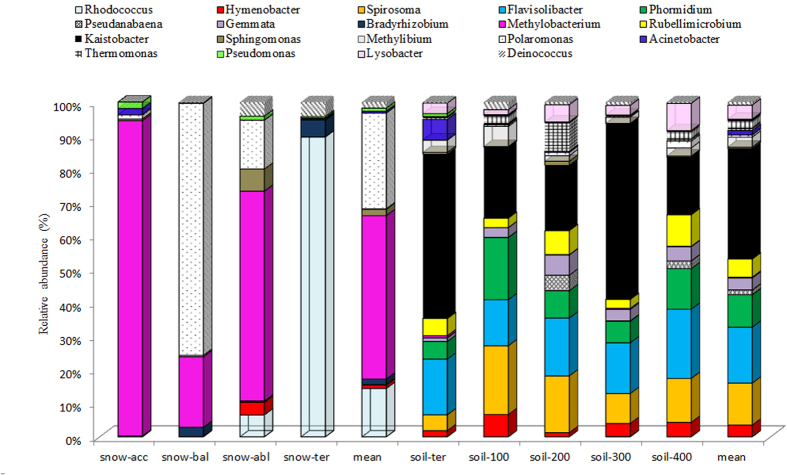
Bacterial community distribution at the genus level in the nine samples.

The sequences that could be classified were assigned to 190 genera. *Acinetobacter*, *Pseudomonas* and *Hymenobacter* were dominant in both glacial snow and glacial soil. Five genera (*Deinococcus*, *Methylobacterium*, *Polaromonas*, *Rhodococcus*, and *Sphingomonas*) were dominant among the four snow samples (Fig. 5), whereas 11 genera (*Flavisolibacter*, *Gemmata*, *Kaistobacter*, *Lysobacter*, *Methylibium*, *Phormidium*, *Rubellimicrobium*, *Spirosoma*, and *Thermomonas*) were dominant among the five soil samples.

Different abundances of bacterial phyla and, to some extent, genera between glacial snow and glacial soil were attributed to environmental conditions. Microorganisms in glacial snow were from aeolian deposition, in which the microbial load from aerosol, dust, and precipitation events directly determined the microbial compositions^15^. Seasonal variations in temperature, nutrient concentration, and solar radiation have the potential to change microbial community composition and to promote the occurrence of species that are well adapted to the glacial snow constraints^16^. In our study, Deinococcus-Thermus, which can endure radiation or high temperature^17^ dominated among the four snow samples. Consistent with this, glacial snow is a harsh but relatively constant environment harbouring a constrained microbial community, which may be particularly well adapted to its environment^18^. Glacier soil, however, is more dynamic in terms of ecosystem development and suffers with primary succession. Gemmatimonadetes is among the most common bacterial groups found in soil and sediment samples from other part of the globe^19^. Armatimonadetes and Planctomycetes are present in other cold habitats^20^,^21^. The dominance of Chloroflexi and Cyanobacteria in glacial soil in our study was consistent with a report demonstrating that photosynthetic autotrophs appeared to be common in all of the soil samples in the Puca glacier^22^. These distributions have been shown to be associated with communities in later stages of ecological succession. Despite these differences, glacial snow and glacial soil shared a few common bacterial phyla with high dominance. Proteobacteria and Actinobacteria, which are the major groups in most freshwater environments around the world, were the most dominant in both glacial snow (Proteobacteria averaged 83.33% and averaged 14.22%) and glacial soil (Proteobacteria averaged 25.75% and averaged 8.12%). Three genera of *Acinetobacter* (0–1.3%), *Pseudomonas* (0–1.1%), and *Hymenobacter* (0–2.2%) were dominant in both glacial snow and glacial soil. These findings were similar to the report of Larose *et al*.^8^, who showed that a common core group of microbial populations existed within snow libraries and meltwater libraries on the Svalbard glacier. In previous studies, *Acinetobacter* was present in East Rongbuk (ER) Glacier^21^, Greenland Ice Sheet Project (GISP 2)^23^, and Taylor Glacier, Antarctica^24^. *Hymenobacter* was present in Gulkana Glacier, Alaska^25^ and Victoria Upper Glacier, Antarctica^26^, and *Pseudomonas* was present in Damma glacier, Central Alps of Switzerland^27^, soil of the Kafni glacier^4^, and the GISP 2 ice core^28^. Thus, the common bacteria present in both glacial snow and glacial soil indicated their wide distribution in the glacial environment.

### Shared and rare orders/families with studies of other sites

For data at the genus and phylum levels, we compared our pyrosequencing results with those of other studies, such as the foreland of Tianshan No. 1 glacier^12^, the High Arctic^29^, the rock-water interface in an East Antarctic freshwater ecosystem, Lake Tawani^30^, and Laohugou No. 12^11^. We also compared our results with those from culture-dependent and culture-independent clone libraries as well as denaturing gradient gel electrophoresis (DGGE), such as ER^16^,^31^, Malan^32^,^33^, Palong^16^, soil of the Kafni glacier^4^, Hailuogou^31^, GISP 2^23^, and the water column at Terra Nova Bay, Antarctica^34^. This comparison indicated that among the 26 phyla, 22 were present in most of these habitats, while four (Candidate division GAL15, Candidate division GN02, Candidate division FBP, and Candidate division MVP-21) were only recovered in our study.

At the genus level, 65 genera from a total of 189 genera were not recovered from the above-mentioned habitats; these genera were as follows: *Acaryochloris*, *Actinoplanes*, *Actinotalea*, *Afifella*, *Agrobacterium*, *Ammoniphilus*, *Anaerococcus*, *Ardenscatena*, *Brevibacterium*, *Caloramator*, *Candidatus Azobacteroides*, *Candidatus Solibacter*, *Capnocytophaga*, *Carnobacterium*, *Chroococcidiopsis*, *Chthonomonas*, *Cloacibacterium*, *Couchioplanes*, *Cupriavidus*, *Dermacoccus*, *Desulfococcus*, *Enhydrobacter*, *Erythromicrobium*, *Erwinia*, *Facklamia*, *Filifactor*, *Fimbriimonas*, *Hylemonella*, *Kaistobacter*, *Klebsiella*, *Kouleothrix*, *Lautropia*, *Leptonema*, *Macrococcus*, *Magnetospirillum*, *Marinobacterium*, *Microbispora*, *Micrococcus*, *Microlunatus*, *Modestobacter*, *Mycoplana*, *Myroides*, *Neisseria*, *Nitriliruptor*, *Oscillochloris*, *Parvibaculum*, *Planomicrobium*, *Pseudanabaena*, *Riemerella*, *Rubrivivax*, *Salinimicrobium*, *Sanguibacter*, *Selenomonas*, *Sporichthya*, *Sporocytophaga*, *Sulfuritalea*, *Tepidimonas*, *Thauera*, *Thermobaculum*, *Thermus*, *Vagococcus*, *Veillonella*, *Vibrio*, *Virgisporangium*, and *Zymomonas*. However, most of the above genera were not dominant in the Chongce glacial environment, except for *Kaistobacter* and *Pseudanabaena*, which were dominant in glacial soil. These latter two genera were photosynthetic bacteria, permitting their survival in a nutrient-limited environment.

### Resource exploitation in the Chongce Ice Cap

In our study, about 26.28–78.67% of the sequences could not be classified into any known genus. This result was consistent with reports from a glacier foreland of the High Arctic^29^ and forest soil^35^. Thus, many of the bacterial phylotypes in the Chongce glacier may be novel. Accordingly, further studies are needed in order to gain a better understanding of the full extent of prokaryotic diversity and its distribution on Earth^29^.

Some known functional bacterial genera were present in the Chongce glacier, including nitrifying bacteria of *Nitrospira*, nitrogen-fixing bacteria of *Bradyrhizobium* and *Rhizobium*, methane-oxidising bacteria of *Methylobacterium* and *Methylopila*, and sulphur- and sulphate-reducing bacteria of *Desulfococcus* (see Supplementary Table S2). *Bradyrhizobium* and *Methylobacterium* were represented by more sequences in glacial snow than in glacial soil, while *Nitrospira* exhibited the opposite distribution pattern. The higher abundance of *Methylobacterium* in glacial snow than in glacial soil was probably due to a prevalence of C1 (such as methanol or formaldehyde) metabolism in snow samples. C1 compound of Formaldehyde is reported to be produced in snowpack^36^ and is a central intermediate in methylotrophic growth. However, we have no direct evidence of the relationship between C1 compound and *Methylobacterium*. Further focused studies using the *mchA* probe are still needed to improve our understanding of the functional bacteria in the glacier. In summary, our data suggested that, despite the potential transfer of cells from ice-marginal habitats, bacterial communities in glacial soil were essentially distinct from those in glacial snow, with most phylotypes occupying distinct niches. Some rare genera and phyla were identified in the Chongce glacier. Although total DNA was obtained from three replicates and mixed to minimise spatial variation, 454 pyrosequencing data from each sampling site were not replicated. Thus, it is impossible to identify statistically different bacterial indicators specialized within different samples using *PCoA,* LEfSe, NMDS, and UPGMA, in order to improve our understanding of spatial succession from glacial snow to glacial soil. Further detailed studies, such as metagenomic analysis, are needed to determine potential drivers for the differences in bacterial diversity and communities between glacial snow and glacial soil based on the chemical properties of snow and soil samples. Metatranscriptomics is also suitable for measuring changes in functional gene expression and their regulation with respect to changing environmental conditions from glacial snow to glacial soil.

## Methods

### Study site and sampling

The Chongce Ice Cap is located on the southern slope of the West Kunlun Mountains, which lie along the northern edge of the Tibetan Plateau close to the Taklimakan Desert. The ice cap extends over a distance of 7 km and contains two prominent domes at 6,530 and 6,374 m^37^,^38^. The elevation of the terminus is about 5,800 m, with an equilibrium line of 5,930 m^39^. A comprehensive field investigation in association with over-year meteorological observations has shown that the Chongce Ice Cap is a summer-accumulation type inland glacier^40^. There is an annual precipitation of 400–450 mm, which is mainly in the form of snowfall^41^. The ice surface temperature ranges from −2 to −3 °C in the summer; however, the temperature has occasionally risen to above 0 °C during the day, providing a small amount of heat to melt the ice. In addition, strong solar radiation can produce a certain amount of ablation. At the local scale, depending on the geomorphology (e.g., slopes and slope breaks), meltwater from our study glacier flowed over bare moraine. Our samples were taken after the melting season in September–October 2013, when meltwater had already inflowed into the soil in the terminus zone.

**Figure 6:**
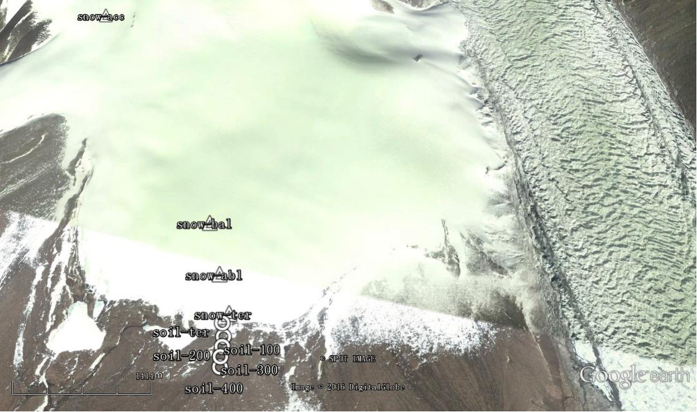
Location of glacial snow and glacial soil sampled in the Chongce Ice Cap.

Glaciers can be divided into two parts based on the equilibrium line: the accumulation zone above the equilibrium line, and the ablation zone below the equilibrium line. The accumulation zone is where material is obtained, whereas the ablation area is where material is expended. The frontal part of the glacier tongue is called the glacier terminus^42^. Snow samples were collected at the terminus zone, ablation zone, balance line, and accumulation zone. Soil samples were collected at the terminus zone and at 100, 200, 300, and 400 m from the terminus zone (Fig. 6). Detailed information regarding the latitude, longitude, and altitude of each sample is given in Supplementary Table S3. Surface samples collected at 0.01 m in depth were discarded, and the underlying snow and soil samples were placed into separate sterile Whirl-Pak bags (Nasco, Salida, CA, USA). For each site, three samples were collected. The methods for extreme care and sample transport were described previously^11^.

### DNA extraction

In a class 100,000 clean room, each of the melted snow samples (300 mL) was filtered through hydrophilic polyethersulfone membranes (Pall; 0.22-μm pore size) with a Millipore *Labscale Tangential Flow Filtration* TFF *System*. The microorganisms on the membranes were eluted by agitation for 2 min with a whirlpool mixer and suspended in 2.0 mL phosphate-buffered saline. Soil samples (5 g) were placed in sterile ceramic mortars after freezing in liquid nitrogen and ground until reaching a powder-like consistency. Total DNA from soil and snow samples was isolated according to the methods described by Zhou *et al*.^43^.

### 16S rRNA gene pyrosequencing

Equal amounts of total DNA extracted from three replicates collected at each point were mixed to minimise spatial variation. Samples were then submitted for pyrosequencing at Personal Biotechnology Limited Company (Shanghai, China). The V1–V3 region of the 16S rRNA gene was selected to construct a community library through tag pyrosequencing. The forward primer of each sample was described in detail previously^11^. The reverse primer was 5′-CCATCTCATCCCTGCGTGTCTCCGACGACTNNNNNNN*TTACCGCGGCTGCTGGCAC*-3′ (the underlined sequence is 454 Life Sciences’ primer A, and the sequence in italics is the broad-range bacterial primer 533R). NNNNNNN designates the unique seven-base barcode used to tag each polymerase chain reaction (PCR) product (see Supplementary Table S3). PCR was carried out with the following conditions: 0.4 μM each primer, 3.2 ng template DNA, 1 × PCR buffer, 0.2 U Q5 Polymerase (M0491S; New England Biolabs [NEB]), and sterile deionised water to a final volume of 25 μL. PCR was performed according to the following thermal cycling conditions: 98 °C for 4 min; 27 cycles at 98 °C for 30 s, 55 °C for 45 s, and 72 °C for 60 s; and a final extension at 72 °C for 7 min.

Fluorescent quantification and emulsion PCR were described previously^11^. Amplicon pyrosequencing was performed on a Roche Genome Sequencer GS FLX^+^ System (Roche Applied Science, Indianapolis, IN, USA). The results of the raw data have been deposited into the NCBI Sequence Read Archive (SRA) under the accession number SRP058670.

### Processing of pyrosequencing data

Data were processed using the Quantitative Insights into Microbial Ecology (QIIME) pipeline^44^. Briefly, sequences that were less than 200 bp or greater than 1,000 bp in length, contained incorrect primer sequences, or contained more than 1 ambiguous base were discarded. Chimeric sequences were checked and deleted using the “align” procedure of the UCHIME algorithm^45^.

### Bioinformatics analysis

Sequence reads from each sample were clustered to give similarity-based OTUs using Cluster Database at High Identity with Tolerance (CD-HIT)^46^ with the minimum sequence identity set to 97%. OTU rarefaction curves, Good’s coverage, and all community richness and diversity indices (ACE, Chao1, Shannon, and Simpson) were generated with Mothur^47^.

A heatmap was constructed using R package vegan to compare the bacterial communities of the most abundant genera in each sample^48^. In addition, PCoA was performed based on the weighted UniFrac distance^49^ in R package vegan, to demonstrate the clustering of different samples.

## Additional Information

**How to cite this article**: Yang, G. L. *et al*. Differences in Bacterial Diversity and Communities Between Glacial Snow and Glacial Soil on the Chongce Ice Cap, West Kunlun Mountains. *Sci. Rep.* **6**, 36548; doi: 10.1038/srep36548 (2016).

**Publisher’s note:** Springer Nature remains neutral with regard to jurisdictional claims in published maps and institutional affiliations.

## Supplementary Material

Supplementary Information

## Figures and Tables

**Table 1:** Observed and estimated bacterial diversity from each sample.

Samples	Raw reads	Valid reads/OTUs	Coverage (%)	Chao1	ACE	Shannon	Simpson
Snow - acc	12821	10805	99.7	115	124	1.511	0.376
Snow - bal	15798	12365	99.7	130	180	1.503	0.348
Snow - abl	13889	10669	99.5	230	271	2.081	0.234
Snow - ter	14485	13434	99.5	235	300	1.175	0.463
Mean	14248	11818	99.6	178	219	1.568	0.355
Soil - ter	15608	12642	98.3	1028	992	5.354	0.016
Soil - 100	14014	8982	95.7	1545	1760	5.673	0.010
Soil - 200	13398	8552	96.6	1291	1203	5.766	0.007
Soil - 300	14668	10794	97.2	1268	1246	5.355	0.021
Soil - 400	12047	7769	95.7	1407	1315	5.783	0.009
Mean	13947	9748	96.7	1308	1303	5.586	0.013

## References

[b1] Hodson A. . Glacial ecosystems. *Ecol*. *Monogr*. 78, 41–67 (2008).

[b2] Edwards A. . Possible interactions between bacterial diversity, microbial activity and supraglacial hydrology of cryoconite holes in Svalbard. ISME J . 5, 150–160 (2011). 20664552 10.1038/ismej.2010.100 PMC3105670

[b3] Amato P. . Bacterial characterization of the snow cover at Spitzberg, Svalbard. *FEMS Microbiol*. *Ecol* . 59, 255–264 (2007). 17328766 10.1111/j.1574-6941.2006.00198.x

[b4] Srinivas T. N. . Comparison of bacterial diversity in proglacial soil from Kafni Glacier, Himalayan Mountain ranges, India, with the bacterial diversity of other glaciers in the world. Extremophiles 15, 673–690 (2011). 21918795 10.1007/s00792-011-0398-8

[b5] Finn D. S., Räsänen K. & Robinson C. T. Physical and biological changes to a lengthening stream gradient following a decade of rapid glacial recession. *Glob*. *Change Biol* . 16, 3314–3326 (2010).

[b6] Liu Y. . Microbial diversity in the snow, a moraine lake and a stream in Himalayan glacier. Extremophiles 15, 411–421 (2011). 21468724 10.1007/s00792-011-0372-5

[b7] Mindl B. . Factors influencing bacterial dynamics along a transect from supraglacial runoff to proglacial lakes of a high Arctic glacier. *FEMS Microbiol*. *Ecol* . 59, 307–317 (2007). 17313580 10.1111/j.1574-6941.2006.00262.x

[b8] Larose C. . Microbial sequences retrieved from environmental samples from seasonal Arctic snow and meltwater from Svalbard, Norway. Extremophiles 14, 205–212 (2010). 20066448 10.1007/s00792-009-0299-2

[b9] Takeuchi N., Koshima S., Yoshimura S., Seko K. & Fujita K. Characteristics of cryoconite holes on a Himalayan glacier, Yala glacier Central Nepal. *Bull*. *Glaciol*. *Res* . 17, 51–59 (2000).

[b10] Grzesiak J. . Microbial community changes along the Ecology Glacier ablation zone (King George Island, Antarctica). Polar Biol . 38, 2069–2083 (2015).

[b11] Zhang S. . Preliminary study on effects of glacial retreat on the dominant glacial snow bacteria in Laohugou Glacier No. 12. *Geomicrobiol*. *J*. 32, 113–118 (2015).

[b12] Wu X. . Bacterial diversity in the foreland of the Tianshan Glacier No. 1, China. *Environ*. *Res*. *Lett*. 7, 1–9 (2012).

[b13] Bahram M., Polme S., Kõljalg U., Zarre S. & Tedersoo L. Regional and local patterns of ectomycorrhizal fungal diversity and community structure along an altitudinal gradient in the Hyrcanian forests of northern Iran. New Phytol . 193, 465–473 (2012). 21988714 10.1111/j.1469-8137.2011.03927.x

[b14] Wang J. J., Soininen J., He J. Z. & Shen J. Phylogenetic clustering increases with elevation for microbes. *Environ*. *Microbiol*. *Rep* . 4, 217–226 (2012). 23757276 10.1111/j.1758-2229.2011.00324.x

[b15] Xiang S. R., Shang T. C., Chen Y. & Yao T. D. Deposition and postdeposition mechanisms as possible drivers of microbial population variability in glacier ice. *FEMS Microbiol*. *Ecol* . 70, 165–176 (2009). 10.1111/j.1574-6941.2009.00759.x 19796140

[b16] Liu Y. . Microbial community structure in moraine lakes and glacial meltwaters, Mount Everest. *FEMS Microbiol*. *Ecol* . 265, 98–105 (2006). 10.1111/j.1574-6968.2006.00477.x 17107422

[b17] Slade D. & Radman M. Oxidative stress resistance in *Deinococcus radiodurans*. *Microbiol*. *Mol*. *Biol*. *Rev*. 75,133–191 (2011). 21372322 10.1128/MMBR.00015-10 PMC3063356

[b18] Wilhelm L., Singer G. A., Fasching C., Battin T. J. & Besemer K. Microbial biodiversity in glacier-fed streams. ISME J . 7, 1651–1660 (2013). 23486246 10.1038/ismej.2013.44 PMC3721114

[b19] Janssen P. H. Identifying the dominant soil bacterial taxa in libraries of 16S rRNA and 16S rRNA genes. *Appl*. *Environ*. *Microb* . 72, 1719–1728 (2006). 10.1128/AEM.72.3.1719-1728.2006 PMC1393246 16517615

[b20] Stres B., Sul W. J., Murovec B. & Tiedje J. M. Recently deglaciated high-altitude soils of the Himalaya: diverse environments, heterogenous bacterial communities and long-range dust inputs from the upper troposphere. PLoS One 8, e76440 (2014). 10.1371/journal.pone.0076440 PMC3784432 24086740

[b21] Liu Y. . Bacterial diversity in the snow over Tibetan Plateau Glaciers. Extremophiles 13, 411–423 (2009). 19159068 10.1007/s00792-009-0227-5

[b22] Nemergut D. R. . Microbial community succession in an unvegetated, recently deglaciated soil. *Microbial*. *Ecol*. 53, 110–122 (2007). 10.1007/s00248-006-9144-7 17186150

[b23] Miteva V. I. & Brenchley J. E. Detection and isolation of ultrasmall microorganisms from a 120,000-year-old Greenland glacier ice core. *Appl*. *Environ*. *Microb* . 71, 7806–7818 (2005). 10.1128/AEM.71.12.7806-7818.2005 PMC1317422 16332755

[b24] Mikucki J. A. & Priscu J. C. Bacterial diversity associated with blood falls, a subglacial outflow from the Taylor Glacier, Antarctica. *Appl*. *Environ*. *Microb* . 73, 4029–4039 (2007). 10.1128/AEM.01396-06 PMC1932727 17468282

[b25] Segawa T., Takeuchi N., Ushida K., Kanda H. & Kohshima S. Altitudinal changes in a bacterial community on Gulkana Glacier in Alaska. Microbes Environ . 25, 171–182 (2010). 21576870 10.1264/jsme2.me10119

[b26] Klassen J. L. & Foght J. M. Characterization of *Hymenobacter* isolates from Victoria Upper Glacier, Antarctica reveals five new species and substantial non-vertical evolution within this genus. Extremophiles 15, 45–57 (2011). 21104190 10.1007/s00792-010-0336-1

[b27] Frey B. . Weathering-associated bacteria from the Damma glacier forefield: physiological capabilities and impact on granite dissolution. *Appl*. *Environ*. *Microb* . 76, 4788–4796 (2010). 10.1128/AEM.00657-10 PMC2901745 20525872

[b28] Miteva V. I., Sheridan P. P. & Brenchley J. E. Phylogenetic and physiological diversity of microorganisms isolated from a deep Greenland glacier ice core. *Appl*. *Environ*. *Microb* . 70, 202–213 (2004). 10.1128/AEM.70.1.202-213.2004 PMC321287 14711643

[b29] Schütte U. M. . Bacterial diversity in a glacier foreland of the high Arctic. *Microb*. *Ecol* . 19, 54–66 (2010). 10.1111/j.1365-294X.2009.04479.x 20331770

[b30] Huang J. P. . Bacterial diversity of the rock–water interface in an East Antarctic freshwater ecosystem, Lake Tawani(P). *Aquat*. *Biosyst* . 9, 4 (2013). 23369372 10.1186/2046-9063-9-4 PMC3740781

[b31] Zhang S., Yang G., Wang Y. & Hou S. Abundance and community of snow bacteria from three glaciers in the Tibetan Plateau. *J*. *Environ*. *Sci*. *China* 22, 1418–1424 (2010). 21174974 10.1016/s1001-0742(09)60269-2

[b32] Xiang S. . Change of bacterial community in the Malan ice core and its relation to climate and environment. *Chinese Sci*. *Bull* . 49, 1869–1875 (2004).

[b33] Xiang S. . Bacterial diversity in Malan ice core from the Tibetan Plateau. Folia Microbiol . 49, 269–275 (2004). 15259767 10.1007/BF02931042

[b34] Giudice A. L. . Marine bacterioplankton diversity and community composition in an Antarctic coastal environment. *Microb*. *Ecol*. 63, 210–223 (2012). 21748267 10.1007/s00248-011-9904-x

[b35] Roesch L. F. . Pyrosequencing enumerates and contrasts soil microbial diversity. ISME J . 1, 283–290 (2007). 18043639 10.1038/ismej.2007.53 PMC2970868

[b36] Kozioł K., Ruman M., Kozak K. & Polkowska Z. Release and transport of toxic, mobile organic compounds (formaldehyde and phenols) on an arctic glacier. APCBEE Procedia 10, 16–20 (2014).

[b37] Chongyi E. . Different responses of different altitudes surrounding Taklimankan Desert to global climate change. *Environ*. *Geol*. 56, 1281–1293 (2009).

[b38] Han J., Masayoshi N., Kumiko G. A. & Lu C. Impact of fine-dust air burden on the mass balance of a high mountain glacier: a case study of the Chongce ice cap, west Kunlun Shan, China. *Ann*. *Glaciol*. 43, 23–28 (2006).

[b39] Zhang W. J., An R. Z., Yang H. A. & Jiao K. Q. Conditions of glacier development and some glacial features in the west Kunlun mountains. *Bull*. *Glacier Res* . 7, 49–58 (1989).

[b40] Ageta Y., Zhang W. J. & Nakawo M. Mass balance studies on Chongce Ice Cap in the West Kunlun Mountains. *Bull*. *Glacier Res* . 7, 37–43 (1989).

[b41] Kang X. C. & Xie Y. Q. The character of the weather and climate in the West Kunlun Mountains area in summer, 1987. *Bull*. *Glacier Res* . 7, 77–81 (1989).

[b42] Wang N. Glacier in English-Chinese Dictionary of Cryospheric Sciences 4 (Meteorology, 2014).

[b43] Zhou J., Bruns M. A. & Tiedje J. M. DNA recovery from soils of diverse composition. *Appl*. *Environ*. *Microb*. 62, 316–322 (1996). 10.1128/aem.62.2.316-322.1996 PMC167800 8593035

[b44] Caporaso J. G. . QIIME allows analysis of high-throughput community sequencing data. *Nat*. *Methods* 7, 335–336 (2010). 20383131 10.1038/nmeth.f.303 PMC3156573

[b45] Edgar R. C., Haas B. J., Clemente J. C., Quince C. & Knight R. UCHIME improves sensitivity and speed of chimera detection. Bioinformatics 27, 2194–2200 (2011). 21700674 10.1093/bioinformatics/btr381 PMC3150044

[b46] Edgar R. C. Search and clustering orders of magnitude faster than BLAST. Bioinformatics 26, 2460–2461 (2010). 20709691 10.1093/bioinformatics/btq461

[b47] Schloss P. D. . Introducing mothur: open-source, platform-independent, community-supported software for describing and comparing microbial communities. *Appl*. *Environ*. *Microbiol*. 75, 7537–7541 (2009). 19801464 10.1128/AEM.01541-09 PMC2786419

[b48] Jami E., Israel A., Kotser A. & Mizrahi I. Exploring the bovine rumen bacterial community from birth to adulthood. ISME J . 7, 1069–1079 (2013). 23426008 10.1038/ismej.2013.2 PMC3660679

[b49] Vazquez-Baeza Y., Pirrung M., Gonzalez A. & Knight R. Emperor: A tool for visualizing high-throughput microbial community data. Gigascience 2, 16 (2013). 24280061 10.1186/2047-217X-2-16 PMC4076506

