# Supplementary material for: Differences in Bacterial Diversity and Communities Between Glacial Snow and Glacial Soil on the Chongce Ice Cap, West Kunlun Mountains
Source: Sci Rep. 2016 Nov 4;6:36548. doi: 10.1038/srep36548 (PMC5109912; doi:10.1038/srep36548)
Supplement: Supplementary Information [file srep36548-s1.doc]

**Supplementary Information**

**Differences** **in Bacterial Diversity and Communities Between Glacial Snow and Glacial Soil on the Chongce Ice Cap, West Kunlun Mountains**

**Guang Li Yang^1*^, Shu Gui Hou^2^, Ri Le Baoge^1^, Zhi Guo Li^3^, Hao Xu^2^, Ya Ping Liu^4^, Wen Tao Du^4^ & Yong Qin Liu^5^**

1. Department of Life Science, Shangqiu Normal University, Shangqiu 476000, China.

2. School of Geographic and Oceanographic Sciences, Nanjing University, Nanjing 210023, China. 3. Department of Environment and Planning, Shangqiu Normal University, Shangqiu 476000, China.

4. Cold and Arid Regions Environmental and Engineering Research Institute, Chinese Academy of Sciences, Lanzhou 730000, China.

5. Key Laboratory of Tibetan Environmental Changes and Land Surface Processes, Institute of Tibetan Plateau Research, Chinese Academy of Sciences, Beijing 100101, China.

*E-mail: guangliyang@163.com


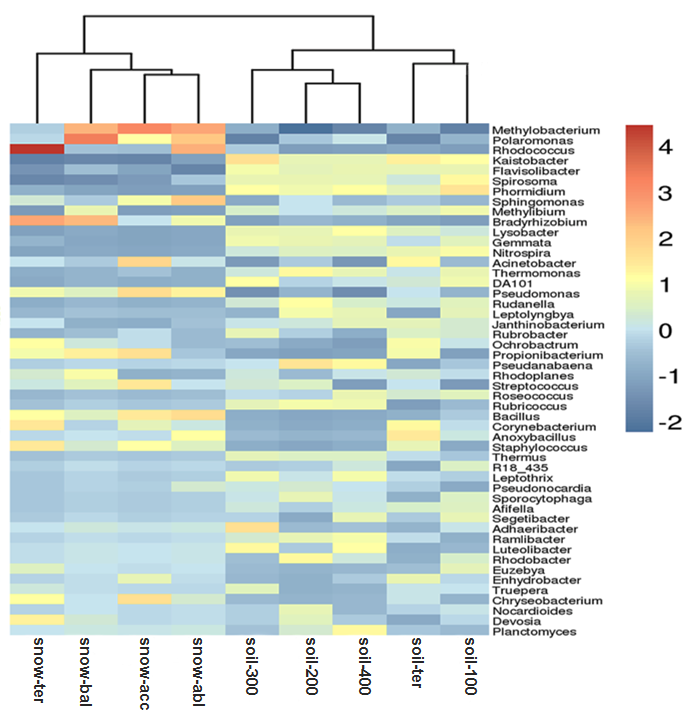


Supplementary Figure S1. Heatmap of the 50 most abundant genera in each sample. The colour intensity in each box indicates the relative percentage of a genus in each sample

**Supplementary Table S1 Bacterial community distribution at the phylum level in the nine samples**

| Taxon | snow-acc | snow-bal | snow-abl | snow-ter | mean | soil-ter | soil-100 | soil-200 | soil-300 | soil-400 | mean |
| --- | --- | --- | --- | --- | --- | --- | --- | --- | --- | --- | --- |
| Acidobacteria | 0.05 | 0 | 0 | 0 | 0.01 | 25.01 | 20.63 | 18.92 | 24.9 | 19.39 | 21.77 |
| Actinobacteria | 16.62 | 1.04 | 8.21 | 30.99 | 14.22 | 11.58 | 12.14 | 5.62 | 5.87 | 5.41 | 8.12 |
| Armatimonadetes | 0 | 0 | 0 | 0 | 0 | 1 | 1.09 | 1.56 | 1.1 | 1.31 | 1.21 |
| Bacteroidetes | 0.36 | 0.11 | 2.35 | 0.3 | 0.78 | 6.86 | 9.53 | 14.1 | 8.75 | 1.11 | 8.07 |
| Chloroflexi | 0 | 0 | 0 | 0.08 | 0.02 | 4.35 | 6.68 | 5.5 | 5.3 | 6.86 | 5.74 |
| Cyanobacteria | 0.02 | 0.06 | 0 | 0.02 | 0.03 | 3.42 | 11.9 | 10.68 | 3.02 | 7.16 | 7.24 |
| Candidate division FBP | 0 | 0 | 0 | 0 | 0 | 0.77 | 2.53 | 2.19 | 4.77 | 3.21 | 2.69 |
| Firmicutes | 1.04 | 0.15 | 0.65 | 0.39 | 0.56 | 1.82 | 0.06 | 0.55 | 0.11 | 0 | 0.51 |
| Gemmatimonadetes | 0.04 | 0 | 0 | 0 | 0.01 | 5.71 | 8.69 | 8.72 | 6.95 | 12.67 | 8.55 |
| Nitrospirae | 0.04 | 0 | 0 | 0 | 0.01 | 1.04 | 1.01 | 0.8 | 0.31 | 0.63 | 0.76 |
| Planctomycetes | 0 | 0 | 0 | 0.08 | 0.02 | 1.6 | 5.2 | 4.37 | 7.21 | 6.46 | 4.97 |
| Proteobacteria | 81.84 | 98.45 | 86.28 | 66.73 | 83.33 | 35.16 | 18.16 | 25.35 | 26.92 | 23.16 | 25.75 |
| Verrucomicrobia | 0 | 0 | 0 | 0 | 0 | 1.28 | 1.21 | 0.74 | 3.63 | 1.1 | 1.59 |
| Deinococcus-Thermus | 0 | 0.17 | 2.48 | 1.38 | 1.01 | 0.17 | 0.56 | 0.41 | 0.52 | 0.32 | 0.40 |

Supplementary Table S2. Numbers of sequences classified within known functional bacterial genera

| Sampling site | Snow - acc | Snow - bla | Snow - abl | Snow - ter | mean | Soil - ter | Soil - 100 | Soil - 200 | Soil - 300 | Soil - 400 | mean |
| --- | --- | --- | --- | --- | --- | --- | --- | --- | --- | --- | --- |
|  | Nitrifying bacteria | | | | | | | | | |  |
| *Nitrospira* | 0 | 0 | 0 | 0 | 0 | 85 | 68 | 26 | 43 | 80 | 60 |
|  | Nitrogen-fixing bacteria | | | | | | | | | |  |
| *Bradyrhizobium* | 2 | 250 | 16 | 214 | 121 | 0 | 2 | 0 | 1 | 0 | 1 |
| *Rhizobium* | 0 | 0 | 0 | 0 | 0 | 1 | 0 | 0 | 0 | 0 | 0 |
|  | Methane-oxidising bacteria | | | | | | | | | |  |
| *Methylobacterium* | 6188 | 1900 | 3952 | 8 | 3012 | 1 | 0 | 9 | 1 | 19 | 6 |
| *Methylopila* | 0 | 0 | 0 | 0 | 0 | 0 | 13 | 0 | 0 | 0 | 3 |
|  | Sulphur- and sulphate-reducing bacteria | | | | | | | | | |  |
| *Desulfococcus* | 0 | 0 | 0 | 0 | 0 | 0 | 0 | 0 | 0 | 0 | 0 |

Supplementary Table S3. Sample information, including latitude, longitude, altitude, and 5′ tagged PCR reversed primers

| Sample | Latitude (N) | Longitude (E) | Altitude (m) | Tagged primer sequence (5′–3′) |
| --- | --- | --- | --- | --- |
| Snow - acc | 35°14′56.04″ | 81°05′28.04″ | 6126.92 | CTAGACA |
| Snow - bal | 35°13′39.15″ | 81°06′54.84″ | 5938.20 | CAGTCTA |
| Snow - abl | 35°13′22.37″ | 81°07′05.86″ | 5871.60 | CAGTACT |
| Snow - ter | 35°13′10.36″ | 81°07′14.63″ | 5832.46 | CAGAGTC |
| Soil - ter | 35°13′06.82″ | 81°07′13.03 | 5800 | CTTGAGT |
| Soil - 100 | 35°13′02.66″ | 81°07′15.17″ | 5789 | CTCACGA |
| Soil - 200 | 35°12′59.52″ | 81°07′15.88″ | 5776 | CTCAGAC |
| Soil - 300 | 35°12′56.26″ | 81°07′16.39″ | 5765 | CTCTCAG |
| Soil - 400 | 35°12′53.11″ | 81°07′17.45″ | 5756 | CTCTGTA |
